# “Superwobbling” and tRNA-34 Wobble and tRNA-37 Anticodon Loop Modifications in Evolution and Devolution of the Genetic Code

**DOI:** 10.3390/life12020252

**Published:** 2022-02-08

**Authors:** Lei Lei, Zachary Frome Burton

**Affiliations:** 1 School of Biological Sciences, University of New England, Biddeford, ME 04005, USA; llei@une.edu; 2 Department of Biochemistry and Molecular Biology, Michigan State University, East Lansing, MI 48824, USA

**Keywords:** elongation factor Tu, evolution of the genetic code, four-way wobbling, genetic code degeneracy, inosine, mitochondria, queuosine, superwobbling, tRNA modification, tRNA wobble U modification

## Abstract

The genetic code evolved around the reading of the tRNA anticodon on the primitive ribosome, and tRNA-34 wobble and tRNA-37 modifications coevolved with the code. We posit that EF-Tu, the closing mechanism of the 30S ribosomal subunit, methylation of wobble U34 at the 5-carbon and suppression of wobbling at the tRNA-36 position were partly redundant and overlapping functions that coevolved to establish the code. The genetic code devolved in evolution of mitochondria to reduce the size of the tRNAome (all of the tRNAs of an organism or organelle). “Superwobbling” or four-way wobbling describes a major mechanism for shrinking the mitochondrial tRNAome. In superwobbling, unmodified wobble tRNA-U34 can recognize all four codon wobble bases (A, G, C and U), allowing a single unmodified tRNA-U34 to read a 4-codon box. During code evolution, to suppress superwobbling in 2-codon sectors, U34 modification by methylation at the 5-carbon position appears essential. As expected, at the base of code evolution, tRNA-37 modifications mostly related to the identity of the adjacent tRNA-36 base. TRNA-37 modifications help maintain the translation frame during elongation.

## 1. Introduction

This review was written to support an interpretation of a confluence of recent and older data. We attempt to bring some simplicity, order and concept to what may seem, at first, like overwhelming complexity and confusion. The genetic code evolved in columns around the structure of the tRNA anticodon. Genetic code columns represent the middle position of the anticodon (tRNA-35), which is and was the easiest anticodon position to read. Initially, tRNA-34 and tRNA-36 were wobble positions, but wobbling was suppressed at tRNA-36, in part, by tRNA-37 modifications. Appreciation of tRNA anticodon loop structure and reading helps to explain genetic code structure and the evolution of tRNA modifications that affect reading of the anticodon. 

Notably, “superwobbling” or four-way wobbling in evolution of the mitochondria has been described and supported by detailed tRNA modification data [1,2,3,4,5]. Phylogenetics indicates pathways of evolution of Archaea, ancient Bacteria, derived Bacteria and Eukarya [5,6]. Evolution of the mitochondria from a bacterial endosymbiont is fundamental to understand evolution of Eukarya [7,8,9,10,11]. Superwobbling indicates the importance of ancient wobble U34 methylation-based modifications at the 5-carbon position. In the mitochondrion, unmodified wobble U34 can potentially read wobble codons ending in A, G, C and U to translate an entire 4-codon sector of the code using a single tRNA species [1,2,5]. At the base of genetic code evolution, however, it appears that tRNA-U34 may often or always have been modified, in part, to suppress superwobbling and to allow evolution of 2-codon sectors [3,4,12,13]. Recent tRNA modification data support this idea. To our knowledge, the relationship of superwobbling to initial genetic code evolution has, for the most part, not been discussed (but see [14]). We posit that 5-carbon U34 methylation-based wobble modifications were essential for the initial evolution of the genetic code. 

Similarly, tRNA wobble adenosine deamination to inosine (tRNA-A34→I34) modifications appear fundamental to the later evolution and enrichment of the code [15,16,17,18,19]. I34, generally, can read wobble codons A, C and U, and the I34 modification is associated with the suppression of synonymous G34 anticodons. G34 is favoured in Archaea and, for the most part, in Bacteria [15]. Put another way, when the I34 wobble modification occurs, the corresponding G34 tRNA anticodon is rarely if ever present. In addition, the introduction of tRNAs with unnatural G34 anticodons in 4-codon boxes can be toxic in Eukaryotes [15,20]. In Bacteria, A34→I34 modification is mostly found for the Arg anticodon (ACG→ICG). By contrast, in Eukarya, the A34→I34 wobble modification is found for Leu (AAG→IAG), Ile (AAU→IAU), Val (AAC→IAC), Ser (AGA→IGA), Pro (AGG→IGG), Thr (AGU→IGU), Ala (AGC→IGC) and, as in Bacteria, Arg (ACG→ICG). Interestingly, in Eukarya, Gly occupies a 4-codon box but does not utilize the A34→I34 modification. We offer two possible explanations below. Because of wobble ambiguity, the A34→I34 modification can only occur in 3- or 4-codon sectors of the genetic code. Some Bacteria encode A34 in 4-codon sectors other than Arg, but, in most of these cases, A34 does not appear to be converted to inosine [16,17]. Because of superwobbling in 4-codon sectors, the A34→I34 modification is not utilized in mitochondria [5]. In response to oxidative and starvation stress, Eukaryotes utilize endonuclease V to cleave I34 tRNAs to stall translation [21].

Bacteria utilize G34→Q34 modifications (Q for queuosine) [5,22,23,24,25,26]. These modifications are found in Eukaryotes, mitochondria and Bacteria but not in Archaea. In Archaea, the queuosine-related modification archaeosine, which involves a homologous enzyme, is found at the G15-position of tRNAs. In humans, queuine is a necessary coenzyme supplied by diet and generated by symbiotic enteric bacteria. Q34 modifications cause more balanced reading of NAU and NAC codons, so the lack of queuosine modifications slows translation [22,23]. Queuosine modifications are only found in column 3 of the genetic code (GUN→QUN anticodons).

Modifications of the anticodon loop tRNA-37 position, just 3’ to the anticodon, also appear to be of importance [15,19,26,27]. TRNA-37 modifications tend to be bulky next to an anticodon U36 or A36 and may help to stabilize intrinsically weaker anticodon-codon interactions. Modifications of tRNA-37 limit frameshifting during translation [27,28,29,30]. TRNA-35 and -36 are rarely modified and are generally read by Watson-Crick pairing to their mRNA codon. We posit that modifications of tRNA-37 help to delimit the anticodon, stabilize base pairing at position 36, stabilize the anticodon-codon interaction, suppress frameshifting [30] and perform other roles, for instance, recognition by aaRS enzymes to charge the cognate tRNA [31]. We find that, as expected, at the base of genetic code evolution, tRNA-37 modifications primarily depend on the adjacent tRNA-36 base, which corresponds to genetic code rows 1–4.

A new tRNA database helps to follow the current trends in the literature [32]. Older databases are also useful [33,34,35,36]. Updated modification data for tRNAs were essential to understand how tRNA modifications affect translation. Some tRNA modifications (i.e., cm^5^U34-based, t^6^A37 and m^1^G37) appear to be as old as the genetic code and, probably, were coevolved with the code and necessary for its initial establishment. Analysis of tRNA modifications at the tRNA-34 and -37 positions strongly supports the hypothesis that the genetic code evolved around the reading of the tRNA anticodon [37,38,39].

The archaeal genetic code is simplest and closest to the code that was present at LUCA (the last universal common (cellular) ancestor). We consider LUCA to be the first membrane-enclosed cells with intact DNA genomes. Pyrococcus furiosis is a reasonable reference organism for an ancient Archaeon and an approximation of LUCA [40,41]. The code is simpler in older bacterial species such as *Thermus thermophilus*, compared to more derived Bacteria, such as *Escherichia coli* and α-Proteobacteria. It appears that the mitochondria were derived from an α-Proteobacteria (Rickettsiales) [5,6,7,10,42,43]. The eukaryotic cytosolic code was derived from Archaea with contributions from an *α*-proteobacterial endosymbiont. Thus, the genetic code can be mostly traced, along with relevant tRNA modification data through evolution of life on Earth [19]. Currently, there is missing tRNA modification data for ancient Bacteria, such as *Thermus thermophilus*. At the time of writing, sequences of only ~5 modified *Thermus thermophilus* tRNAs have been reported out of a total of about 47 tRNAs. At the time of writing, no *Thermus thermophilus* tRNA with a modified or unmodified U34 has yet been reported [32]. Combining these missing data with this paper would be a useful contribution.

Aminoacyl-tRNA synthetases (aaRS) attach cognate amino acids to the 3′-ends of tRNAs [31,37,44]. Evolution of aaRS enzymes has been described in detail. AaRS are of the two incompatible folding classes I and II with structural subclasses A→E. The class II aaRS GlyRS-IIA was refolded into a class I aaRS (probably a primitive ValRS-IA). In addition to their incompatible fold, class I aaRS have an in-phase N-terminal extension relative to class II aaRS. The class II aaRS mounts the enzyme active site on a surface of antiparallel β-sheets. By contrast, the class I aaRS mounts the enzyme active site at the C-terminal ends of a set of parallel β-sheets. GlyRS-IIA (glycine aminoacyl-tRNA synthetase; class II; structural subclass A) is the root of all aaRS enzymes. In ancient Archaea, GlyRS-IIA is a sequence homolog of ValRS-IA and IleRS-IA. Tracing the evolution of aaRS enzymes describes the evolution of the genetic code. The genetic code evolved from Archaea to ancient Bacteria to more derived Bacteria. Eukarya are a fusion of multiple Archaea and multiple Bacteria probably involving a number of endosymbionts and/or other large horizontal gene transfers [6,10,45]. We find that a simple narrative for the evolution of life on Earth is obtained by comparing genetic codes, tRNA-34 and tRNA-37 modifications, aaRS and tRNAome data from a small number of reference organisms.

## 2. Evolution of the Genetic Code around the tRNA Anticodon

**Figure 1:**
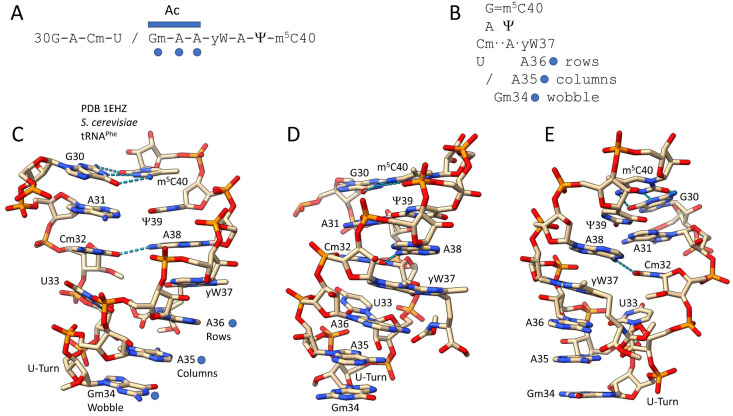
The Saccharomyces cerevisiae tRNA^Phe^ anticodon loop (PDB 1EHZ). (**A**) The linear sequence is shown. The anticodon (Ac) is indicated (3 blue dots). (**B**) The folded loop structure is shown. / indicates a U-turn. (**C**–**E**) Three views of the anticodon loop are shown. The anticodon is indicated in C (blue dots). Blue dashed lines indicate H-bonds. Colors: (beige) carbon; (blue) nitrogen; (red) oxygen; (orange) phosphorous. Abbreviations: (cm) 2′-*O*-methylcytidine; (Gm) 2′-*O*-methylguanosine; (yW) wybutosine (modification of G); (Ψ) pseudouridine; (m^5^C) 5-methylcytidine.

In Figure 1, the Saccharomyces cerevisiae tRNA^Phe^ anticodon loop is shown (PDB 1EHZ) [46]. In Figure 1A, the linear modified sequence is shown. In Figure 1B, the folded structure is indicated. Figure 1C–E are three orientations of the anticodon loop structure including part of the anticodon stem. The genetic code evolved around the structure of the tRNA anticodon. The anticodon triplet is tRNA positions 34, 35 and 36. TRNA-34 is the wobble position at which diverse wobble contacts to mRNA codons are allowed, adjusted and tuned in evolution. TRNA-35 is the central position, which represents genetic code columns and is the easiest position for the translation system to read. TRNA-36 represents genetic code rows 1–4. Generally, the tRNA-35 and -36 positions are read during translation as Watson—Crick base pairs versus the mRNA codon. As in Saccharomyces cerevisiae tRNA^Phe^, tRNA-35 and -36 are generally unmodified.

A detailed and rational model for pre-LUCA evolution of the genetic code has been published [37,38,39]. The genetic code is highly structured and more simply structured in Archaea than in other organisms. Most evolution is in code columns, which represent the tRNA-35 base. For instance, in column 1 (tRNA-35A), related hydrophobic amino acids Val, Met, Ile and Leu are found, and these chemically similar amino acids are added to their cognate tRNAs by ValRS-IA, MetRS-IA, IleRS-IA and LeuRS-IA, which are closely related aaRS class IA enzymes. Similarly, in column 2 (tRNA-35G), amino acids Thr, Pro and Ser are found. Thr and Ser are closely related amino acids, and ThrRS-IIA, ProRS-IIA and SerRS-IIA are closely related aaRS class IIA enzymes. The code is proposed to have evolved through stages. Initially, both tRNA anticodon positions 34 and 36 were wobble positions, at which only 2-assignments (purine versus pyrimidine) were possible. Wobbling was suppressed at position 36 by evolution of EF-Tu, the 16S rRNA “latch” (i.e., G530~A1492 and A1493; *Thermus thermophilus* numbering) [47,48] and modifications of anticodon loop position 37. Suppression of wobbling at position 36 allowed the code to expand from 8-amino acids (complexity 2 × 4) to a maximum complexity of 32-assignments (complexity 2 × 4 × 4). Because of fidelity mechanisms, the standard genetic code froze at 20-amino acids plus stops.

The primordial sequence of the 7-nt anticodon loop was close to 32-CU/BNNAA-38 (/ indicates a U-turn; B = G, C or U (not A); N = A, G, C or U). In Figure 1, four bases (30, 31, 39 and 40) that are normally part of the anticodon stem are also shown. The G30 = m^5^C40 base pair is evident. The expected A30→Ψ39 base pair was disrupted by the pseudouridine rearrangement, perhaps to adjust the conformation and dynamics of the loop. Typically, the loop includes a U-turn after U33. A U-turn is a U-shaped turn in the RNA backbone [49]. The U-turn loop conformation is important to present the 3-nt anticodon (tRNA-34, -35 and -36). The Cm32~A38 H-bond can be characterized as a weak reverse Hoogsteen pair Cm32 (O2)→A38 (N6). This interaction is thought to regulate the U-turn geometry and dynamics of the anticodon loop [19,26]. The yW37 (wybutosine) modification of G is a bulky modification that is thought to stabilize interactions of the A36 anticodon base with its cognate codon and also to suppress frameshifting during translation. 

## 3. Evolution of Life on Earth

A simple narrative for evolution of life on Earth is proposed in which LUCA evolved to Archaea [41,50]. As a reference organism that is close to LUCA, we propose Pyrococcus furiosis that has a tRNAome that is very similar in sequence to tRNA^Pri^ (a primordial tRNA) [40]. We propose that Archaea evolved to ancient Bacteria, such as *Thermus thermophilus*. We selected *Thermus thermophilus* because it has a simple but intact tRNAome. Unfortunately, the reported tRNA modification data for *Thermus thermophilus* is not complete at the time of writing. As a model organism for more derived Bacteria, we relied mostly on *Escherichia coli*. If data were available, we would incorporate the closest bacterial relative of the eukaryotic mitochondria. *Escherichia coli*, however, appears to be a reasonable model, albeit with several differences from the endosymbiont that became the mitochondria. We support the hypothesis that eukaryotic mitochondria were derived from an α-proteobacterial endosymbiont within an Asgard Archaea [11,51]. Eukaryotes, however, arose as a complex set of genetic fusions of multiple Archaea and multiple Bacteria. For the purposes of this paper, we trace tRNA U34, A34→I34 and G34→Q34 modifications through evolution. We discuss maintenance of the Ile-Met sector. Maintenance of 1-codon sectors (i.e., for Met and Trp) in evolution was difficult and was abandoned during evolution of mitochondria [5]. We consider modifications of anticodon position 37 [19,52]. We combine these data with evolution of aaRS enzymes and analyses of tRNAomes. To our knowledge, these issues have largely not been raised or have not been integrated in this manner in published papers. We consider our presentation to be highly informative to describe the major advances in evolution of the genetic code through the natural biological history of Earth.

## 4. Ancient Archaea

**Figure 2:**
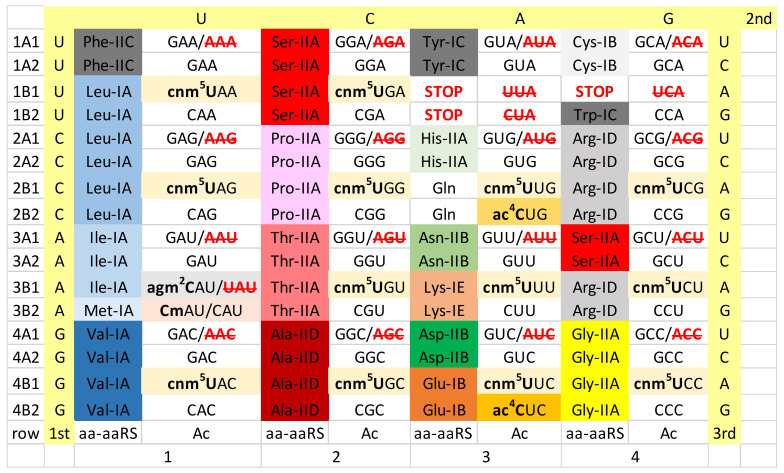
The genetic code in Archaea (i.e., Pyrococcus furiosis). Genetic code columns (tRNA-35) are labelled 1–4. The leftmost table column gives row designations. Row 1–4 numbers indicate the tRNA-36 base. Codon bases (1st, 2nd, 3rd) are shaded pale yellow. TRNA-34 bases are indicated with modifications in bold type. Amino acids and aaRS structural classes and subclasses are shown (i.e., Phe-IIC indicates tRNA^Phe^ is charged by PheRS-IIC) (aa-aaRS). GAA/**AAA** indicates anticodon (Ac) data. Anticodon GAA reads codons UUU and UUC, and anticodon AAA is not utilized. Color highlighting is meant to emphasize particular table features and evolution of aaRS enzymes through Earth’s history in Figures 2–6. Data were modeled on Pyrococcus furiosis but tRNA modification data are not complete, so some data were inferred or utilized from other Archaea. Color shading is meant to be largely consistent in Figures 2–6.

In this paper, we present or approximate the genetic codes of several reference organisms including some related data. Figure 2 shows an approximation of the Pyrococcus furiosis genetic code. Because of missing tRNA modification data, some information has been taken from or inferred from other Archaea. At the time of writing, significant tRNA modification data is available for Pyrococcus furiosis, Methanocaldococcus jannachii, Methanococcus maripauludis, Sulfolobus acidocaldarius and Haloferax volcanii [3,4,12]. The genetic code is presented as a 64-assignment code. Codon sequence surrounds the table. Anticodon data is enriched with tRNA modification data mostly for the wobble base (tRNA-34). The amino acid and structural class (class I or II; structural subclasses A–E) of the aminoacyl tRNA synthetase (aaRS) enzymes was included. Anticodons that are not utilized in an organism or domain may be shown in red with strikethrough. To follow the narrative of this paper, all of these data are necessary to consider in order to compare genetic codes relevant to the generation of Eukarya and mitochondria.

First of all, A34, in which A is unmodified, is rarely or never allowed in Archaea [15]. Rather, in Archaea, G34 appears to always be utilized. As a wobble base, G34 has the advantage of pairing with codon wobble U, as a G~U wobble pair, or else with codon wobble C, as a Watson-Crick G = C pair. At the base of code evolution, U34 appears to seldom or never be unmodified, specifically by a methylation-based modification at the 5-carbon of U34 (cm^5^U34-based modifications). For the precise chemistry of tRNA modifications, please refer to the Modomics Database [26,33,34,35,36]. We propose that cm^5^U-based modifications (i.e., cnm^5^U in Pyrococcus furiosis) suppress superwobbling, which is observed for 4-codon sectors in mitochondrial tRNAs [1,2,5]. A cnm^5^U34 tRNA, therefore, is likely confined to read codon wobble A and G. Superwobbling, by contrast, would allow unmodified U34 to read A, G, C and U, which would prevent evolution of 2-codon sectors. To evolve 2-codon genetic code sectors (i.e., for columns 1, 3 and 4), therefore, required cm^5^U-based modifications. 

Furthermore, 1-codon sectors were difficult to evolve and maintain. Consider the Ile/Met 4-codon sector, in which Met occupies a 1-codon (AUG) sector. We posit that the 4-codon Ile/Met sector was originally a 4-codon Ile sector that Met invaded, eliminating the Ile UAU anticodon [37,38,39]. In Archaea and Bacteria, Ile utilizes a CAU anticodon. In some Archaea, C34 is modified to 2-agmatidine (agm^2^C) to read codon AUA (Ile) but not codon AUG (Met) [4,53,54,55]. Note that a cnm^5^UAU anticodon would read both AUA (Ile) and AUG (Met), causing miscoding. Met utilizes two tRNAs, tRNA^Met^ (i.e., CmAU) for elongation and tRNA^iMet^ (i.e., unmodified CAU) for initiation. A very similar strategy is utilized to maintain the 1-codon Met box in most or all prokaryotes [26,53,56,57,58,59]. The Trp 1-codon sector (UGG) is read by the Trp anticodon CCA that is specific for codon UGG. The UCA anticodon is not utilized, because Trp shares a 2-codon box with a stop codon (UGA) that is recognized by a protein release factor that binds to the mRNA UGA stop codon to terminate translation on the ribosome [60]. Anticodon cnm^5^UCA would read codons UGA and UGG, causing miscoding and suppressing translation stops. This explains why Trp utilizes anticodon CCA, rather than cm^5^UCA, to read codon UGG. 

GlnRS-IB was a eukaryotic innovation that was transferred from Eukarya to Archaea and Bacteria by horizontal gene transfer [51,61]. Some archaeal and bacterial species, therefore, lack GlnRS-IB and instead use GluRS-IB to convert tRNA^Gln^ to Glu-tRNA^Gln^. In these organisms, an amidotransferase converts Glu-tRNA^Gln^ to Gln-tRNA^Gln^ for translation [62,63]. So, GlnRS-IB in Archaea and Bacteria was a later acquisition in evolution (i.e., perhaps ~1.5 to 2.5 billion years ago). In Archaea, GluRS-IB, LysRS-IE and GlnRS-IB (from Eukarya) are closely related aaRS enzymes [37,38,39]. In some cases, the historic structural subclassifications for aaRS are deceptive. LysRS-IE is more closely related to GluRS-IB and GlnRS-IB than any of these three aaRS enzymes are to CysRS-IB. Similarly, AspRS-IIB, AsnRS-IIB and HisRS-IIA are reasonably closely related aaRS enzymes. We posit that a pre-LUCA AspRS-IIA evolved to AspRS-IIB to suppress tRNA charging errors, before evolution of AsnRS-IIB from AspRS-IIB. These homologies create a striped pattern of aaRS relatedness in column 3, indicative of the mode by which column 3 sectored [37,38,39]. The striped pattern in Archaea is somewhat disrupted by evolution of LysRS-IIB in Bacteria to replace archaeal LysRS-IE.

## 5. Ancient Bacteria

**Figure 3:**
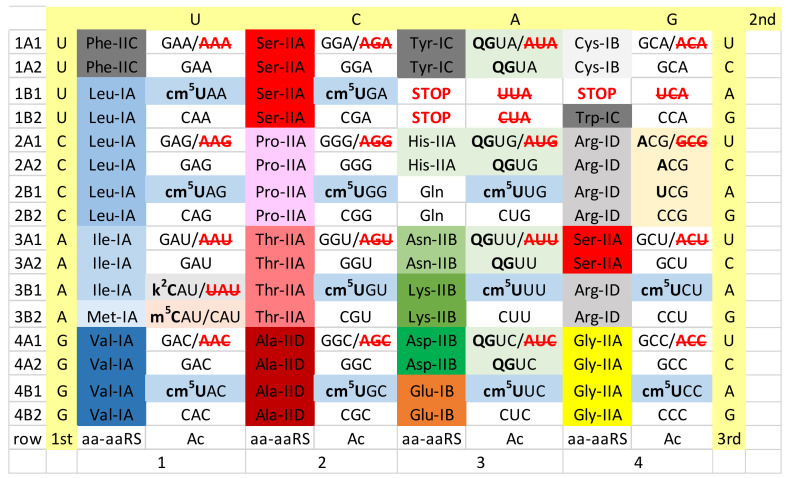
The genetic code in ancient Bacteria (i.e., *Thermus thermophilus*). GAA/**AAA** indicates anticodon GAA is utilized and AAA is not, to encode Phe. **QG**UA/**AUA** indicates the G34→Q34 modification and AUA is not utilized. LysRS-IIB is a bacterial innovation. **cm^2^U**AA for Leu indicates that the precise 5-carbon U modification to suppress superwobbling is not currently reported for *Thermus thermophilus*. Some tRNA modification data were inferred by identifying enzymes in *Thermus thermophilus*. It is not clear to us at the time of writing how the Arg 4-codon box is read.

As a model organism for an ancient Bacterium, we selected *Thermus thermophilus* (Figure 3). Unfortunately, to date, there is too much missing tRNA modification data for *Thermus thermophilus*, so, perhaps, the analysis we present can be refined in the future. Although data are currently missing, we posit a 5-carbon cm^5^U34-based modification to suppress superwobbling and to support the existence of 2-codon genetic code sectors. In column 4, the Arg 4-codon sector may be an intermediate in evolution of the A34→I34 modification. *Thermus thermophilus* tRNA^Arg^ encodes anticodon ACG and lacks a tRNA with a GCG anticodon. *Thermus thermophilus*, however, appears to lack the enzyme expected to convert A34→I34 (tRNA adenosine deaminase). Currently, we do not know whether an unknown modification of A34 is present in *Thermus thermophilus*. If present, unmodified Arg (UCG) would read the entire 4-codon box. Modified anticodon cm^5^UCG would be expected to read CGA and CGG Arg codons. Anticodon CCG reads the CGG Arg codon. Precisely how *Thermus thermophilus* reads the Arg 4-codon box, therefore, does not appear to be currently reported. It is possible that *Thermus thermophilus* represents an intermediate stage in evolution of the Arg (ACG→ICG) anticodon present in most Bacteria [15]. 

In column 1, the Ile/Met sector is maintained in much the same manner as in Archaea, although, using a slightly different modification. In *Thermus thermophilus*, tRNA lysidine (34) synthetase (TilS) is present, so it appears *Thermus thermophilus* utilizes the 2-lysidine Ile (k^2^CAU) modification [26,53,56,57,58]. The 2-lysidine modification is chemically similar to the 2-agmatidine modification in Archaea. 2-lysidine is utilized to read Ile codon (AUA) but not Met codon (AUG). The UAU anticodon is not utilized, because cm^5^U34 would read both codons AUA (Ile) and AUG (Met). The elongator tRNA^Met^ (CAU) has a lightly modified C34 (i.e., CmAU). As in Archaea, the initiator tRNA^iMet^ (CAU) is unmodified. 

**Figure 4:**
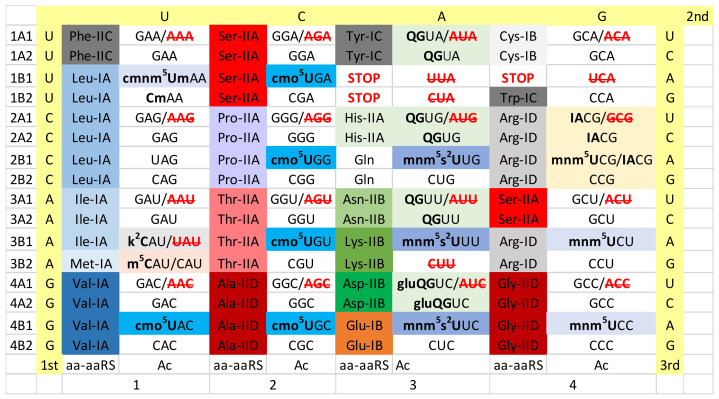
The genetic code in derived Bacteria (i.e., *Escherichia coli*). Innovations include: (1) ProRS-IIA takes on additional bacterial features; (2) Arg ACG→ICG/**GCG** is utilized (*Thermus thermophilus* appears to lack tRNA adenosine deaminase); and (3) GlyRS-IIA can be replaced in some Bacteria by GlyRS-IID. As in *Thermus thermophilus*, LysRS-IIB and type II tRNA^Tyr^ are utilized. This table is based on incomplete tRNA modification data. *Escherichia coli* appears not to utilize Lys anticodon CUU.

In column 3, *Thermus thermophilus* utilizes a type II tRNA^Tyr^, with a longer V-loop (14-nt; the primordial length of the type II V-loop) [64]. *Thermus thermophilus* TyrRS-IC interacts with the V-loop tip as a determinant in Tyr placement to form Tyr-tRNA^Tyr^. Although the corresponding tRNAs have not been analyzed for modifications, *Thermus thermophilus* encodes enzymes for queuosine modification of column 3 tRNAs. Bacterial LysRS-IIB replaces archaeal LysRS-IE. LysRS-IIB is derived in evolution from AspRS-IIB, probably by duplication and repurposing of the gene copy [37]. So, even when an aaRS enzyme is replaced by a very different aaRS in evolution (i.e., LysRS-IE (Archaea)→LysRS-IIB (Bacteria)), evolution of the replacement aaRS may arise within the same column (column 3). Replacement of archaeal LysRS-IE with bacterial LysRS-IIB breaks the striped pattern observed for the simpler archaeal genetic code (compare Figure 2 and Figure 3, column 3). We posit that Archaea, which have a simpler genetic code, are older organisms than Bacteria (compare Figure 2 and Figure 3) [41,65]. *Thermus thermophilus* has a GlyRS-IIA and a ProRS-IIA that lacks an editing active site, similar to GlyRS-IIA and ProRS-IIA in Archaea. Later in bacterial evolution, GlyRS-IID and ProRS-IIA (i.e., sometimes with an added editing active site) evolved. More derived Bacteria utilize CmoA and CmoB enzymes to generate the cmo^5^U modification found in 4-codon sectors in columns 1 and 2 of the *Escherichia coli* genetic code (i.e., Val, Ser, Pro, Thr and Ala) (Figure 4). *Thermus thermophilus* lacks a detectable CmoA or CmoB homolog. Some Rickettsiales utilize CmoA and CmoB, but many do not. In mitochondria, unmodified U34 (superwobbling) is utilized to read 4-codon sectors. Also, CmoA and CmoB were probably missing in the bacterial endosymbiont that became the mitochondria. 

## 6. Derived Bacteria

Because of available tRNA modification data, our model organism for a more derived Bacterium is generally *Escherichia coli* (Figure 4) [32]. In this regard, we would prefer to also show full information for the nearest relative of the α-proteobacterial species (i.e., Rickettsiales) that became the mitochondria, but we cannot identify these data. Also, because of horizontal gene transfers, a modern Rickettsiales might not be an apt comparison to the mitochondria. We posit that the 5-carbon of U34 is often modified in Bacteria to suppress superwobbling and to maintain 2-codon sectors. TRNA-34 modification data tend to evolve in columns, as might be expected for enzymes that bind the tRNA anticodon to add a modification. Columns represent the central position tRNA-35 of the anticodon.

Interestingly, in columns 1 and 2, the cmo^5^U34 modification is found in tRNAs encoding Val, Ser, Pro, Thr and Ala [26,66,67]. The cmo^5^U34 modification, therefore, is found in 4-codon sectors and was expected to read codons ending in wobble A, G and U but not C. For tRNA^Pro^ (cmo^5^UGG); however, this single tRNA^Pro^ (cmo^5^UGG) supports viability of Salmonella, indicating that cmo^5^U34 anticodons can potentially read the entire Pro 4-codon box. In Bacillus subtilis, tRNA^Leu^ (UAG), in which U34 appears to be unmodified, may utilize superwobbling [32].

In column 4, tRNA^Arg^ (ACG→ICG), encoded A34 is modified to inosine (I34) by deamination [15,16,17]. Interestingly, tRNA^Arg^ (GCG), which is favoured in Archaea, is not utilized. When A34 is converted to I34, the corresponding G34 anticodon is not utilized. Anticodon I34 reads codon wobble bases U, C and A but not G. To read the 4-codon Arg box, tRNA^Arg^ (ICG), (mnm^5^UCG) and (CCG) are utilized. TRNA^Arg^ (mnm^5^UCG) probably reads codons CGA and CGG. Also, in column 4, GlyRS-IIA may be replaced with GlyRS-IID in some derived Bacteria (i.e., *Escherichia coli*). In α-Proteobacteria, GlyRS-IIA is utilized, as in *Thermus thermophilus* and Archaea. Not surprisingly, GlyRS-IID is utilized in plant chloroplasts (i.e., from Cyanobacteria), although GlyRS-IIA, not GlyRS-IID, is utilized in the plant mitochondria [51].

In column 1, the Ile/Met 4-codon sector is essentially as described for Archaea and ancient Bacteria. Ile anticodon GAU reads codons AUU and AUC. Ile anticodon k^2^CAU (k^2^C for 2-lysidine modification of C) reads codon AUA (Ile) but not AUG (Met) [26,53,56]. Anticodon UAU is not utilized because even a cm^5^UAU would read both AUA (Ile) and AUG (Met) causing miscoding. Met utilizes tRNA^Met^ (m^5^CAU) (elongator Met) and tRNA^iMet^ (unmodified CAU) (initiator Met). Maintaining 1-codon sectors presents problems. For instance, in mitochondria, Ile and Met occupy 2-codon sectors to minimize the size of the tRNAome and its supporting proteome [5].

In column 3, queuosine modification for G34 (G34→Q34) is utilized [24,25,26]. Interestingly, the G34→Q34 column 3 modification is passed forward into the eukaryotic cytosol and also into mitochondria. All G34 anticodons in column 3 are modified G34→Q34. There can be further modification of queuosine to glutamyl-queuosine (tRNA^Asp^ (gluQGUC)). As in *Thermus thermophilus*, tRNA^Tyr^ is a type II tRNA with a longer V-loop. As expected, this bacterial feature of tRNA^Tyr^ goes forward to the mitochondria but not the eukaryotic cytosol. LysRS-IIB is utilized in most Bacteria in place of archaeal LysRS-IE. *E. coli* appears to lack tRNA^Lys^ (CUU). Apparently, tRNA^Lys^ (mnm^5^s^2^UUU) reads both Lys codons AAA and AAG, as expected.

## 7. Mitochondria 

**Figure 5:**
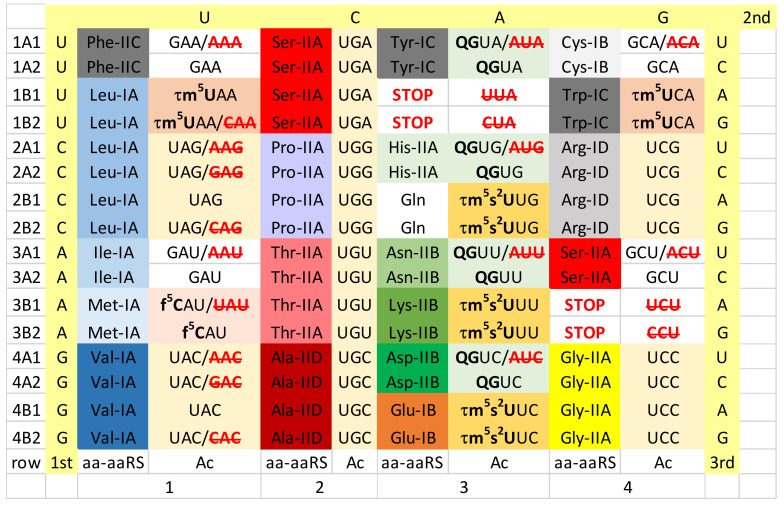
The genetic code in human mitochondria. A major strategy to shrink the mitochondrial tRNAome was superwobbling (beige shading). In mitochondria, Met, Ile and Trp utilize 2-codon sectors. The distribution of stop codons has changed. GlnRS-IB is not imported into human mitochondria. G34→Q34 modifications are utilized in column 3. τ indicates taurine modifications. Many unused anticodons were not struck out in this figure (except in column 1). It appears that the human mitochondrial code may be completely and accurately reported [5].

Mitochondria were evolved from an α-proteobacterial endosymbiont, perhaps a Rickettsiales. The genetic code for human mitochondria is shown in Figure 5 [5]. Because of human health issues, better tRNA modification data are available for human mitochondrial tRNAs than for most Eukarya. Furthermore, human mitochondria utilize only 22-tRNAs, so humans, vertebrates and animals have a significantly reduced mitochondrial tRNAome. We believe the data shown in Figure 5 are essentially complete and accurate.

The main strategy for shrinking the mitochondrial tRNAome is “superwobbling” or 4-way wobbling, in which a single unmodified U34 tRNA reads an entire 4-codon box [1,2,5]. This strategy is used for all 4-codon boxes, including 4-codon boxes encoding Leu, Val, Ser, Pro, Thr, Ala, Arg and Gly (beige shading in Figure 4). In column 3, G34→Q34 modifications are utilized (light green shading in Figure 5). 2-codon boxes with U34 utilize a modified U34, as expected, to restrict superwobbling, which would cause miscoding. Evolution of specific modifications generally aligns in columns, as expected. Human mitochondria include no 1-codon sectors (i.e., to encode Met and Trp) [5]. Instead, atypically, 2-codon sectors are utilized for Ile, Met and Trp. Because a stop codon (UGA) was lost in forming a Trp 2-codon sector, the loss was compensated by converting AGG and AGA, which in Bacteria are Arg codons, into mitochondrial stop codons. Human mitochondria do not import GlnRS-IB. Instead, GluRS-IB is utilized to synthesize Glu-tRNA^Gln^, which is converted to Gln-tRNA^Gln^ by an amidotransferase. The bacterial mitochondrial ancestor did not encode GlnRS-IB, which was a eukaryotic innovation transferred to Archaea and Bacteria by horizontal gene transfers [51]. Archaeal Pyrococcus furiosis also lacks GlnRS-IB and uses a similar tRNA^Gln^ charging strategy. Mitochondria utilize LysRS-IIB, which was derived initially from a bacterial source. Not all mitochondrial and chloroplast tRNAomes, tRNA modifications and collections of aaRS enzymes are the same, so human mitochondria are an example without complete generality.

## 8. The Eukaryotic Cytosol 

**Figure 6:**
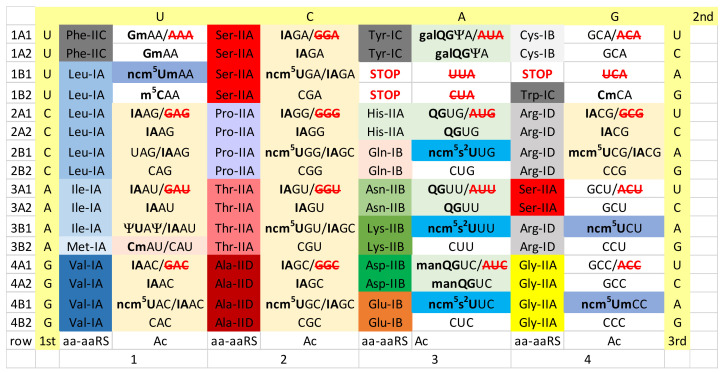
The genetic code in the eukaryotic cytosol (i.e., human). Shading and symbols are as in Figure 2, Figure 3, Figure 4 and Figure 5. **ΨU**AΨ indicates ΨAΨ (Ψ for pseudouridine).

In the eukaryotic cytosol, the genetic code reflects the fusion of an Asgard Archaea and the α-proteobacterial endosymbiont that became the mitochondria [6,8,9,10,11] (Figure 6). A major feature in evolution of the eukaryotic cytosol is the expansion of the A34→I34 strategy (beige shading in Figure 6). All 4-codon sectors except that encoding glycine utilize the A34→I34 modification and, also, suppression of the corresponding G34 anticodon [15]. We suspect that the Gly 4-codon sector did not adopt the A34→I34 modification strategy because of evolutionary pressures to adjust rates of translation. It appears that the Gly GCC anticodon may have been better balanced with the mnm^5^UCC and CCC anticodons. Although *Escherichia coli* does not do this, some Bacteria encode A34 in 4-codon sectors other than Arg (ACG→ICG), but, generally, in these cases, A34 does not appear to be converted to inosine [15,17]. To prevent miscoding, the A34→I34 modification strategy can only occur in 3-(Ile) or 4-codon sectors, because I34 recognizes codon wobble bases U, C and A.

In column 1, the Ile/Met 4-codon sector underwent some eukaryotic cytosol-specific changes. The Ile anticodon AAU→IAU modification is utilized, allowing the reading of Ile codons AUU, AUC and AUA. Also, in Eukaryotes, anticodon UAU→ΨAΨ (Ψ for pseudouridine) can be used to read codon AUA (Ile) but not AUG (Met) [32]. In Prokaryotes, generally, UAU is not utilized even with modification (Figure 2, Figure 3 and Figure 4). In column 3, G34 is modified to Q34 or a modified Q34 (i.e., galactosyl- or mannosyl-queuosine) [24,25]. Because queuosine in column 3 is a bacterial innovation, the eukaryotic cytosol takes on significant bacterial characteristics in the genetic fusion(s) that resulted in eukaryogenesis. LysRS-IIB is another bacterial innovation that is utilized in the eukaryotic cytosol. Apparently, LysRS-IE, derived from an Asgard archaeal partner in the fusion, was rejected. GlyRS-IIA could be derived from an Asgard Archaea, an α-Proteobacteria or by horizontal gene transfer from another archaeal or bacterial source.

The eukaryotic cytosol does not utilize the cmo^5^U34 modification found in some Bacteria but not others (columns 1 and 2; compare Figure 4 and Figure 6). Probably, the cmo^5^U34 modification was absent in the bacterial endosymbiont that became the mitochondria. We posit that optimal balanced reading of 4-codon boxes may be tuned by coevolution of tRNA sequences and anticodon modifications. Therefore, the cmo^5^U34 modification may be more compatible paired with synonymous G34 anticodons, as observed in *Escherichia coli* for Val, Ser, Pro, Thr and Ala (Figure 4). By contrast, in Eukarya, the ncm^5^U34 modification may be more compatible paired with isoacceptor I34 anticodons (Figure 6). This could help explain why Gly utilizes anticodons GCC (rather than ICC, which does not appear to be utilized), ncm^5^UmCC and CCC anticodons in Eukarya (Figure 6). The ncm^5^UmCC Gly anticodon probably is restricted to read Gly codons GGG and GGA.

## 9. Sources of Eukaryotic and Mitochondrial aaRS Enzymes

Table 1 reflects work in progress toward understanding how human cytoplasmic and mitochondrial aaRS enzymes may have evolved through the complex genetic fusion(s) that generated Eukarya [51]. The story is tangled because of 1) (sometimes multiple) horizontal gene transfers; 2) multiple archaeal and bacterial contributions to the eukaryotic genetic make-up; 3) eukaryotic genetic innovations; and 4) coevolution of cytosolic and mitochondrial tRNAs and aaRS enzymes. A recent paper describes molecular events associated with eukaryogenesis [11]. Generally, cytosolic tRNAs are thought to have archaeal origins and mitochondrial tRNAs probably have an α-proteobacterial origin. Interestingly, tracing mitochondrial aaRS to α-proteobacterial origins has been challenging, indicating many diverse bacterial contributions to Eukarya evolution [61,68,69]. In plants, several aaRS enzymes are co-targeted to the mitochondria and the chloroplasts, and chloroplast aaRS, in some cases, appear to have been derived from a cyanobacterial source [69]. Also, there are apparent discrepancies relating to the proteobacterial sourcing of mitochondrial aaRS [61,68,69]. A full and reliable accounting of the sourcing of aaRS enzymes in the eukaryotic cytosols (i.e., animals and plants) and in mitochondria and chloroplast organelles does not appear to yet be available. Also, nearest apparent bacterial relatives of most mitochondrial and chloroplast aaRS have not been unambiguously reported [51].

Mitochondrial aaRS enzymes are encoded within the eukaryotic cell nucleus. For two aaRS, the gene encoding the cytoplasmic aaRS and the mitochondrial aaRS is the same (GlyRS-IIA (GARS) and LysRS-IIB (KARS)). In most cases, by contrast, separate genes encoding the cytoplasmic and mitochondrial aaRS are utilized (Table 1). Mitochondrial aaRS enzymes are expected to include a mitochondrial targeting sequence. We conclude the following. Many cytosolic eukaryotic aaRS enzymes appear to be bacterial in origin (i.e., seven cytosolic aaRS enzymes: AlaRS-IID (AARS), ArgRS-ID (RARS), AspRS-IIB (DARS), IleRS-IA (IARS), LysRS-IIB (KARS), ThrRS-IIA (TARS) and ValRS-IA (VARS)). In the cases in which there are separate cytoplasmic and mitochondrial aaRS genes, the cytoplasmic aaRS gene is likely to have an archaeal origin and the mitochondrial gene invariably appears to have a bacterial origin (i.e., AsnRS-IIB (NARS and NARS2), GluRS-IB (EPRS and EARS2), HisRS-IIA (HARS and HARS2), LeuRS-IA (LARS and LARS2), MetRS-IA (MARS and MARS2); PheRS-IICα and PheRS-IICβ (FARSA, FARSB and FARS2), ProRS-IIA (EPRS and PARS2), SerRS-IIA (SARS and SARS2), TrpRS-IC (WARS and WARS2) and TyrRS-IC (YARS and YARS2)). In human cells, EPRS is a hybrid gene encoding both GluRS-IB and ProRS-IIA. Twelve cytosolic aaRS enzymes appear to have an archaeal origin (i.e., 12 cytosolic aaRS enzymes: AsnRS-IIB (NARS), CysRS-IB (CARS), GluRS-IB (EPRS), GlyRS-IIA (GARS), HisRS-IIA (HARS), LeuRS-IA (LARS), MetRS-IA (MARS), PheRS-IICα/β (FARSA and FARSB), ProRS-IIA (EPRS), SerRS-IIA (SARS), TrpRS-IC (WARS) and TyrRS-IC (YARS)). The CARS gene appears to have split into cytosolic CARS and mitochondrial CARS2 by gene duplication and divergence. As noted above, GlnRS-IB is not imported into human mitochondria. In the eukaryotic cytosol, GlnRS-IB appears to be a eukaryotic innovation that was transferred to Bacteria and Archaea by multiple horizontal gene transfers [51,61]. Some cytosolic aaRS genes appear to have undergone multiple horizontal gene transfers. Examples include AlaRS-IID (AARS), AsnRS-IIB (NARS), ArgRS-ID (RARS), CysRS-IB (CARS), HisRS-IIA (HARS), MetRS-IA (MARS), ProRS-IIA (EPRS) and TyrRS-IC (YARS). Because of complex genetics, horizontal gene transfers and divergent evolution, there may be significant differences comparing eukaryotic cytosols, mitochondria and chloroplasts from very different species. It appears that for the first eukaryotes to have survived may have required multiple and complex horizontal gene transfers and/or multiple endosymbioses.

## 10. TRNA Modifications Are as Old as LUCA

**Figure 7:**
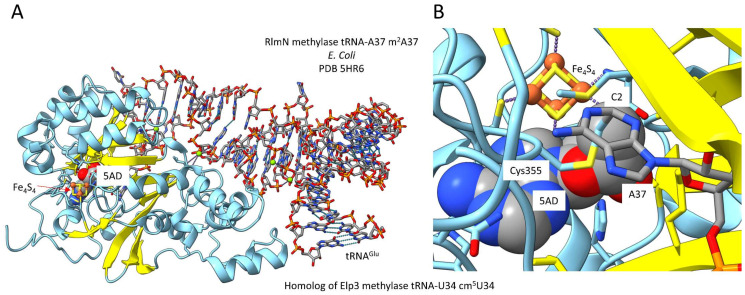
Elp3 (tRNA-cm^5^U34 methyl transferase) is an ancient enzyme. The Elp3 homolog RlmN (tRNA-m^2^A37) methylase is shown. (**A**) A view of the RlmN structure. (**B**) A detail and rotated view. β-sheets are yellow. The Fe_4_S_4_ cage is indicated. A 5′-deoxyadenosine (5AD) radical is formed from S-adenosylmethionine (space-filling representation). The radical reaction mechanism of RlmN methylase involves a covalent intermediate linking Cys355 and m^2^A37. In Archaea, Elp3 may function somewhat differently. Enzymes of this class include an S-adenosylmethionine methylase domain and a lysine acetyl transferase homology domain that binds acetyl coenzyme A.

We consider Pyrococcus furiosis to be a reasonable reference organism for LUCA. Pyrococcus furiosis includes an Elp3 homolog that may encode tRNA-U34 cm^5^U methylase that initiates the cnm^5^U34 modification (Figure 2). The Elp3 enzyme class is as ancient as LUCA. These enzymes utilize S-adenosylmethionine, an iron-sulphur complex, acetyl coenzyme A and radical intermediates to methylate the 5-carbon of U34 [70,71,72]. The cm^5^U34 reaction appears to include multiple steps and cooperation of the S-adenosylmethionine and the lysine acetyltransferase homology (coenzyme A-binding) active sites. S-adenosylmethionine is converted to a 5’deoxyadenosine radical. Acetyl-CoA is bound in the lysine acetyltransferase homology domain. An acetyl radical may then be formed and attached at the C5 position of U34. In Figure 7, the related *Escherichia coli* enzyme RlmN methylase is shown that modifies the 2-carbon of tRNA-A37 [73,74]. The RlmN images were selected because they better emphasize some properties of these ancient enzymes. The image in Figure 7B is a detail and different orientation than that shown in Figure 7A. The (β−α)_6_ partial barrel that binds S-adenosylmethionine was derived from a (β−α)_8_ TIM barrel (TIM for triose phosphate isomerase). The partial barrel domain is identified by 6-parallel β-sheets with intervening α-helices in an open barrel shape. These ancient enzymes include a linked lysine acetyltransferase homology active site. The coenzyme A-binding region of the lysine acetyltransferase homology domain is identified in the image by antiparallel β-sheets (Figure 7A). Because Elp3 homologs are older than LUCA, TIM barrels, S-adenosylmethionine, Fe_4_-S_4_ cages, lysine acetyltransferases, coenzyme A and cm^5^U34-based modifications must be older than LUCA [75,76]. We posit that cm^5^U34-based tRNA modifications, which were required to form 2-codon genetic code sectors, were required to evolve the genetic code, which must also be older than LUCA. Because modifications of the tRNA-37 position were important or essential to read the tRNA-36 position, we posit that t^6^A37 and m^1^G37 modifications are likely older than LUCA (see below).

## 11. TRNA-37 Modifications

**Figure 8:**
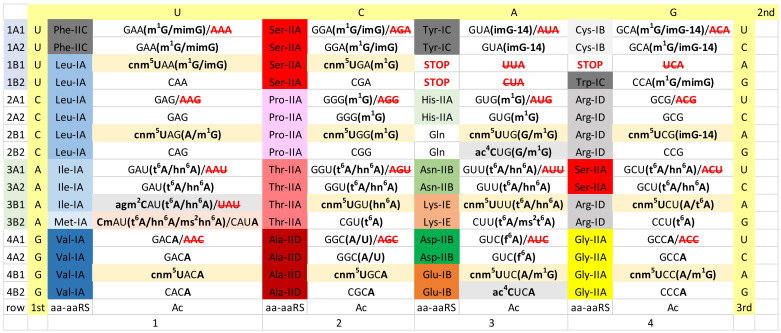
TRNA-37 modifications in Archaea. The tRNA-34 and tRNA-37 modifications are indicated in bold type. TRNA-37 modifications track the tRNA-36 position (rows 1–4). Row 1 (light blue) and 3 (light green) numbers are shaded for emphasis.

To gain potential insights into tRNA-A37 and -G37 modifications, we visualized the genetic code for Archaea along with reported tRNA-37 modifications (Figure 8). We strongly support the idea that Archaea are the most ancient organisms on Earth and the most similar to LUCA [41,50,65]. Because of missing data, we combined results for tRNA-37 modifications from a number of archaeal species. We conclude the following. At the base of genetic code evolution, the major determinant of tRNA-37 modifications was the identity of the tRNA-36 base. As a result, similar or identical tRNA-37 modifications tend to cluster in genetic code rows (rows 1–4). This result makes sense because tRNA-36 and tRNA-37 are adjacent bases. The most-bulky ancient tRNA-37 modifications (i.e., t^6^A37 and hn^6^A37) are associated with tRNA-U36 (row 3) indicating that U36 may have required stabilization during early code evolution. TRNA-m^1^G37 modifications appear important or essential for reading tRNA-A36 (row 1) [27]. Of course, in principle, the identity of tRNA-37 could relate to the reading of the first codon position in mRNA instead of the tRNA-36 position, but we do not favor this idea. It appears to us that mRNA evolution generally chased tRNA evolution and that the genetic code evolved around the tRNA anticodon and the anticodon delimiting base tRNA-37. Also, tRNA-37 modifying enzymes can read the tRNA-36 base directly but not the complementary codon base. Throughout row 3 (tRNA-U36), tRNA-37 t^6^A, hn^6^A and ms^2^hn^6^A are found. One exception is tRNA^iMet^, for which the anticodon loop is unmodified. From this comparison, it appears to us that tRNA-37 modifications may be most important to support translation elongation rather than to support initiation. Further discrimination of tRNA^Ile^ (CAU), tRNA^Met^ (CAU) and tRNA^iMet^ (CAU) is evident in the acceptor stems of the tRNAs [37].

According to tRNA anticodon preference rules, the genetic code evolved around the tRNA anticodon. At the wobble position tRNA-34, G was favored over C/U. At anticodon positions tRNA-35 and tRNA-36, the preference rules are C>G>U>>>A, and preferences are much stronger for the tRNA-36 position, which, early in code evolution, was a wobble position [37,38,39]. In keeping with these rules, unmodified tRNA-A37 appears favorable for row 4 (tRNA-C36), and C is the most favored tRNA-36 base (Figure 8). Although data are missing, it appears that tRNA-37 modifications can also be absent for row 2 (tRNA-G36). By contrast, in Archaea, row 3 (tRNA-U36) appears to be the most heavily modified for tRNA-37. We posit that tRNA-t^6^A37 may be among the most ancient row 3 modifications. Notably, t^6^A37 and hn^6^A37 are large N-6 modifications of A37 that may be important for stabilization of tRNA-U36 during translation elongation [27]. Row 1, tRNA-A36, was the last row to fill during evolution of the genetic code. Row 1 is modified for tRNA-37. We posit that tRNA-m^1^G37 may be the most ancient row 1 modification. Because m^1^G37 (row 1) appears to be a smaller modification than t^6^A or hn^6^A37 (row 3), we posit that tRNA-A36 may have been easier to stabilize than tRNA-U36 after suppression of tRNA-36 wobbling (i.e., by EF-Tu, 30S ribosomal closing and tRNA-37 modifications). Also, there is the difference in the identity of the t^6^A37 and m^1^G37 bases. Removing the tRNA-m^1^G37 modification increases the frameshifting of a near-cognate tRNA in the ribosome P-site [30].

Preference rules for the tRNA anticodon may also partially explain why the glycine 4-codon sector did not evolve the A34→I34 modification in Eukaryotes. According to anticodon preference rules, Gly (GCC) is the most favored anticodon in the genetic code [37,38,39]. This may partly explain why the unmodified GCC anticodon was favored over a modified ICC anticodon for the glycine 4-codon sector in Eukarya. Consideration of anticodon preference rules appears to reinforce our model for evolution of the genetic code, our interpretations of tRNA anticodon loop modifications and our hypothesis that the genetic code evolved around the reading of the tRNA anticodon on the primitive pre-LUCA ribosome.

## 12. Partial Redundancy and Overlap in Translation Functions

**Figure 9:**
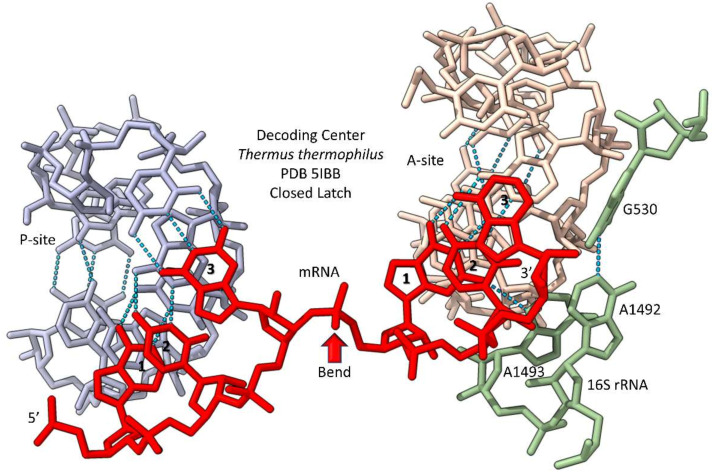
The decoding center of the *Thermus thermophilus* ribosome during peptide bond synthesis [78]. Colors: (grey) P-site tRNA anticodon loop; beige) A-site tRNA anticodon loop; sea (green) the “latch”; and (red) mRNA. A bend in the mRNA that separates the P-site and A-site codons and anticodons is indicated (red arrow). Codon positions (1, 2 and 3) and the 5’→3’ directionality of the mRNA are indicated.

Because of their ancient evolution and central importance to life, very early, translation systems evolved overlapping, partly redundant and mutually-reinforcing systems. Such redundancy and overlap are observed in: (1) translational fidelity and frame maintenance; (2) tRNA sequence and modification; and (3) aaRS enzyme selectivity in tRNA charging. Because translation systems were central to life and evolution of the genetic code, functional redundancy and, also, backed-up, resilient functions were necessary to evolve stable systems. On the ribosome, translational accuracy and maintenance of the translation frame appear to be partially reinforcing systems. Specifically, translational accuracy and frame maintenance involve: (1) EF-Tu GTPase; (2) the 16S rRNA “latch” (30S ribosomal subunit closing mechanism); (3) a mRNA bend between the P-site and A-site codons; and (4) modifications of the tRNA-37 base [30,47,48]. EF-Tu is the most important factor in translational accuracy. EF-Tu binds the aminoacylated tRNA (aa-tRNA) and docks it on the ribosome. If the tRNA anticodon-mRNA codon interaction is cognate, EF-Tu hydrolyzes GTP to close the conformation of the ribosome 30S subunit (also referred to as closing the 16S rRNA latch). Once the latch is closed, EF-Tu releases the cognate A-site aa-tRNA to accommodate into the peptidyl transferase center for peptide bond transfer. Accommodation requires a surprisingly large motion of the 3′-end of the aa-tRNA. Figure 9 shows a detail of a catalytic ribosome structure (PDB 5IBB) with the P-site (peptidyl-site) and A-site (aminoacyl-site) tRNAs [77,78]. To avoid confusion, only the decoding center is shown in the image, not the peptidyl transferase center, and only the anticodon loops of the P-site and A-site tRNAs are shown. The 16S rRNA latch (G530~A1492 and A1493; *Thermus thermophilus* numbering) is shown in its closed conformation. The mRNA bends between the P-site and A-site codons. The bend (or “kink”) orients the 3′-ends of the tRNAs in the peptidyl transferase center, but the bend also separates the P-site and A-site tRNA anticodons in the decoding center [79,80,81]. Separation of the P-site and A-site anticodons in the decoding center has multiple effects. First, the bend in the mRNA prevents collision of the two anticodon loops. Notably, without the bend, A-site tRNA-37 might collide with the P-site tRNA. Second, separation of the P-site and A-site tRNAs helps the tRNAs to maintain the translation frame by acting as ratchet pawls. Closing the latch maintains the accuracy of translation by confirming the codon-anticodon interaction but also helps to maintain the frame. Modifications at the tRNA-37 position help to delineate the A-site anticodon and to maintain the translation reading frame. Notably, mutations that disable tRNA-37 modifications can cause slippage of the translation frame [30]. Bulky 37 modifications are associated most strongly with U36 (row 3) and A36 (row 1) anticodons, indicating that, among other features, tRNA-37 modifications help to read otherwise less stable codon–anticodon interactions (Figure 8) [27].

The tRNA anticodon loop has a highly specialized sequence with modifications that affect anticodon readout and loop dynamics (Figure 1). Also, the anticodon loop is a target for multiple interactions with modifying enzymes and the cognate aaRS. Thus, any particular sequence or modification can have multiple purposes and interactions. Mutations, therefore, can have complex and unanticipated effects. The anticodon immediately follows a U-turn following a U, in the 7-nt anticodon loop. The primordial tRNA anticodon loop sequence was close to 32-CU/BNNAA-38 (/ indicates a U-turn; B indicates G, C or U (not A); N indicates any base) [37,38,39,82]. Modifications are common at positions 32, 34, 37 and 38 [19,26,27]. A weak interaction (i.e., a C~A reverse Hoogsteen pair) is often observed between positions 32 and 38. The C32~A38 interaction may help to preserve the U-turn loop conformation that is important to maintain the codon-anticodon interaction. So, tRNA anticodon loop modifications, sequences and dynamics are evolved features that affect translational accuracy and output. We consider anticodon loop features to be complex, with overlapping inputs and outputs (i.e., sequences and modifications) that are evolved for different species and for individual tRNAs. 

Matching a cognate tRNA to its cognate aaRS is also a problem with multiple inputs [31]. Notably, aaRS enzymes may read: (1) the discriminator base (XCCA-3’; X is the discriminator); (2) the acceptor stem; (3) the anticodon loop; (4) the tRNA elbow (where the D loop and the T loop interact); (5) expanded V-loops in type II tRNAs; and (6) tRNA modifications. We posit that aaRS recognition of their cognate tRNA, therefore, is a product of multiple partially overlapping determinants and anti-determinants. Table 1 indicates how cognate tRNAs and aaRS enzymes may have been sorted after genetic fusion of multiple Archaea and multiple Bacteria to form Eukarya. 

## 13. Conclusions

We strongly support the model that the genetic code evolved around the reading of the tRNA anticodon on the primitive pre-LUCA ribosome [37,38,39]. Analyses of modifications at the tRNA-34 and -37 anticodon loop positions support this concept. Suppression of wobbling at the tRNA-36 position was essential to evolve the code.

**Figure 10:**
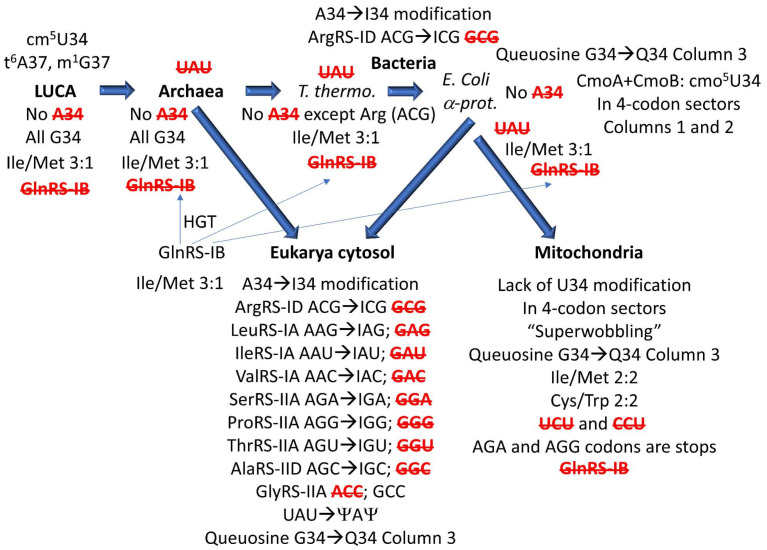
Evolution of tRNA-34 wobble modifications. Superwobbling in mitochondria indicates that cm^5^U34-based modifications were necessary to generate 2-codon sectors to evolve the LUCA code. Red strikethrough indicates that an anticodon is not utilized. Ψ indicates pseudouridine. In mitochondria, 2-codon sectors are utilized to encode Ile, Met and Trp. HGT indicates horizontal gene transfer. Not all anticodon strike-outs are listed for superwobbling in mitochondria.

Some of the conclusions of this paper are shown schematically in Figure 10. The presentation in this paper was partly organized around work of others [19,26]. We wished to expand the previous presentations to make it easier for non-experts in tRNA modification and anticodon readout to shape a detailed understanding. We also wanted to emphasize the problem of code evolution and devolution in mitochondria as an evolutionary milestone that helps explain ancient pre-LUCA evolution and also eukaryogenesis [5]. Figure 10 indicates that, in outline, evolution of life on Earth was simple with a small number of main branches. We advocate for the model that LUCA evolved first to Archaea. Archaea gave rise to Bacteria [41,50,65]. Fusion of an Asgard Archaea and an α-Proteobacteria (i.e., Rickettsiales) gave rise to Eukarya, with division and establishment of separate and partly overlapping translation systems for the eukaryotic cytosol and the mitochondria [6,10,45]. Many other archaeal and bacterial genetic inputs were likely during eukaryogenesis, but, at the time of writing, these other gene transfers are somewhat less completely understood (Table 1) [51].

We consider analysis of the evolution of genetic codes and tRNA-34 modifications through Earth’s history to support our narrative (Figure 2, Figure 3, Figure 4, Figure 5 and Figure 6). The simplest genetic code is that of Archaea (Figure 2), indicating that Archaea is closest to LUCA [41,50,65]. Generally, unmodified A34 is not allowed in Archaea, and only G34 is utilized. This fact alone indicates how genetic code degeneracy evolved. Degeneracy evolved through natural processes of the evolution of the reading of the tRNA anticodon on the primitive ribosome. To evolve the genetic code, universal or near universal cm^5^U34-based modifications were necessary to suppress superwobbling (4-way wobbling) and to, thus, support evolution of 2-codon genetic code sectors. Lacking 2-codon sectors, the genetic code would have been limited to a maximum of 16-amino acids. 

Translation systems evolved through ancient bacteria to more derived bacteria. To date, too much tRNA modification data remains unreported for *Thermus thermophilus*. The missing *Thermus thermophilus* data will enhance this discussion. More derived Bacteria are genetically diverse with many innovations. In some derived bacteria, G34 anticodons in 4-codon boxes pair with the cmo^5^U34 modification (Val, Ser, Pro, Thr and Ala), unmodified UAG (Leu) and mnm^5^UCC (Gly) (Figure 4). The emergence of the A34→I34 modification is relevant. The A34→I34 innovation is associated with suppression of the otherwise preferred G34 anticodon (Figure 6). The A34→I34 modification expanded in Eukarya. In 3- and 4-codon boxes, I34 anticodons may partner with particular U34 modifications (i.e., ncm^5^U34 and mcm^5^U34, in Eukarya). The G34→Q34 (Q for queuosine) modification in genetic code column 3 arose in derived Bacteria and was transmitted to the eukaryotic cytosol and to mitochondria. 

Tracing the evolution of the Ile/Met 4-codon sector through evolution is instructive. Maintaining 1-codon sectors for Met and Trp in the genetic code required proteome support. Probably, for this reason, mitochondria abandoned 1-codon sectors (Figure 5) to simplify the tRNAome and its supporting proteome [5]. In prokaryotes, we posit that Met invaded a 4-codon Ile sector during genetic code evolution, suppressing use of the UAU anticodon and resulting in C34 modifications to read Ile (i.e., CAU→agm^2^CAU and k^2^CAU). The 2-agmatidine modification of C34 found in Archaea and the related 2-lysidine modification in Bacteria read codon AUA (Ile) but not codon AUG (Met). In Eukarya, the Ile anticodon modification (UAU→ΨAΨ) arose, rescuing Ile anticodon UAU.

We posit that 4-codon sectors of the genetic code were balanced using different evolved strategies in different organisms to utilize, generally, 3-isoacceptor tRNAs to read 4-codons. This balance was mostly achieved by adjusting use of G34 or A34-derived and U34 anticodons. In Archaea, G34 and cm^5^U34-based anticodons (i.e., cnm^5^U34) were utilized (Figure 2). In some derived Bacteria, G34 and cmo^5^U34 anticodons were partnered for columns 1 and 2 of the code (4-codon sectors). In column 4, anticodon ICG partners with mnm^5^UCG to encode Arg, and GCC partners with mnm^5^UCC to encode Gly (Figure 4). According to anticodon preference rules, Gly (GCC) is expected to be the most favoured anticodon in the genetic code. Gly (GCC) is associated with unmodified tRNA-A37 in Archaea (Figure 8), possibly reflecting the preferred anticodon GCC status. In Eukarya, diverse strategies were evolved for balancing 3- and 4-codon sectors (Figure 6). Very clearly, anticodons that are not utilized in organisms are very important for maintaining balanced reading of tRNAs (Figure 2, Figure 3, Figure 4, Figure 5 and Figure 6). In mitochondria, 4-codon sectors utilize a single tRNA with unmodified U34 to read the entire 4-codon box, indicating that small mitochondrial genome size was more important than optimization of balancing multiple tRNAs for the most rapid and efficient translation of the 4-codon sectors (Figure 5).

We posit that the genetic code evolved around the reading of the tRNA anticodon on the primitive pre-LUCA ribosome. Analysis of tRNA wobble modifications strongly supports the idea that the genetic code evolved around the reading of the anticodon wobble position. Code degeneracy arose from wobbling at the 34 and 36 positions, as previously described [37,38,39]. Wobbling limits coding to pyrimidine-purine discrimination, so, only 2-assignments were possible at a tRNA wobble position. Thus, evolving 1-codon sectors posed difficulties with miscoding and anticodon ambiguity. TRNA-37 modifications evolved to help lock down the anticodon tRNA-36 position, in part, to suppress wobbling at position 36. Also, wobbling at tRNA-36 was suppressed by evolution of EF-Tu and the 16S rRNA latch (Figure 8 and Figure 9). Analysis of how the genetic code devolved in evolution of the mitochondria strongly supports these views. We do not find the concept of late wobbling evolution to be credible [14,83]. We posit that the genetic code evolved and sectored largely around the reading of tRNA wobble positions.

Column 3 of the genetic code is split entirely into 2-codon sectors. We have posited that initially column 3 was divided into alternating 2-codon Asp and Glu sectors [37,38,39]. Our model explains the striped pattern of related aaRS enzymes in Archaea column 3 (Figure 2). According to our model for code evolution, tRNA-U34 modification (i.e., cm^5^U34) may have been necessary to suppress superwobbling at tRNA-U34 and to achieve the 8-amino acid fractionation of the code. According to our model, therefore, cm^5^U34-based modifications may have been necessary to achieve a genetic code including 8-amino acids. Alternatively, only tRNAs with 34-GU-35 (Asp) and 34-CU-35 (Glu) may have initially been utilized. In this case, C34 may have required modification to read mRNA wobble 3A. We conclude that tRNA wobble modifications appear to have been necessary as early as at the 8-amino acid stage of genetic code evolution.

The model we support for evolution of life on Earth is a fairly well-accepted model (Figure 10). The analysis we present, therefore, appears to be straightforward and reasonable. Our work with the initial evolution of the genetic code is also very consistent with our current analysis [37,38,39]. As noted, the analyses that we present will be enhanced by the acquisition of additional tRNA modification data. 

We imagine eukaryogenesis proceeding through a tense evolutionary bottleneck from FECA to LECA (first to last eukaryotic common ancestors). It appears to us that eukaryogenesis was tortured, involving many endosymbiotic and other large horizontal gene transfer events, only some of which resulted in identified eukaryotic organelles. Apparently, contributions were made to the process by many archaeal and many bacterial genes and, also, the genetic fusions were balanced by many compensating eukaryotic innovations [11]. The FECA to LECA bottleneck is reflected in the evolution of aaRS enzymes through eukaryogenesis (Table 1) [51]. Clearly, genes were transferred between many different organisms, including the horizontal transfer of the gene encoding GlnRS-IB from Eukarya to Archaea and Bacteria.

## 14. Future work

Specific goals for future work include: (1) obtain additional tRNA modification data (i.e., for Pyrococcus furiosis and *Thermus thermophilus*); (2) Improve the data underlying Table 1 (obtain optimal aaRS enzyme evolutionary sourcing for: (1) animals; (2) plants; (3) mitochondria; and (4) chloroplasts); (3) improve the description of evolution of tRNA-34 modifications and modification enzymes; and (4) improve the description of evolution of tRNA-37 modifications and modification enzymes. These additional data would enhance the narrative presented here. Mitochondria were an older acquisition than chloroplasts in evolution of Eukaryotes. A more-detailed model for the more recent evolution of chloroplasts (i.e., tRNAs, tRNA modifications, aaRS enzymes and genetic code), therefore, would enhance the understanding of the acquisition of mitochondria and the evolution of Eukaryotes through endosymbiosis.

## Figures and Tables

**Table 1:** Human aaRS enzymes (and genes) in the cytosol and mitochondria. PMW indicates Parvarchaeota, Micrarchaeota, and Woesearchaeota [51]. The mitochondria utilize GluRS-IB to generate Glu-tRNA^Gln^ and a transamidase to generate Gln-tRNA^Gln^ for translation. Abbreviations: Cyto) cytoplasmic; Mito) mitochondrial.

aaRS	Cyto	Cyto/Mito	Mito
AlaRS-IID	AARS (Bacteria)		AARS2 (Bacteria)
ArgRS-ID	RARS (Bacteria)		RARS2 (Bacteria)
AsnRS-IIB	NARS (Archaea)		NARS2 (Bacteria)
AspRS-IIB	DARS (Deinococcus-Thermus; Bacteria)		DARS2 (Bacteria)
CysRS-IB	CARS (Archaea)		CARS2 (Archaea)
GlnRS-IB	QARS (Eukarya)		Transamidation
GluRS-IB	EPRS (PMW; Archaea)		EARS2 (Bacteria)
GlyRS-IIA		GARS (Euryarchaeota; Archaea)	
HisRS-IIA	HARS (Archaea)		HARS2 (Bacteria)
IleRS-IA	IARS (Lentisphaera; Bacteria)		IARS2 (Bacteria)
LeuRS-IA	LARS (PMW; Archaea)		LARS2 (Bacteria)
LysRS-IIB		KARS (Bacteria)	
MetRS-IA	MARS (Archaea)		MARS2 (Bacteria)
PheRS-IIC	FARSA + FARSB (Euryarchaeota?; Archaea)		FARS2 (Bacteria)
ProRS-IIA	EPRS (Archaea)		PARS2 (Bacteria)
SerRS-IIA	SARS (TACK; Archaea)		SARS2 (Bacteria)
ThrRS-IIA	TARS (i.e., Gemmatimonadetes?; Bacteria)		TARS2 (Bacteria)
TrpRS-IC	WARS (PMW; Archaea)		WARS2 (Bacteria)
TyrRS-IC	YARS (Archaea)		YARS2 (Bacteria)
ValRS-IA	VARS (Deltaproteobacteria?; Bacteria)		VARS2 (Bacteria)

## Data Availability

Not applicable.
